# Socio-psychological factors associated with anticipated stigma toward COVID-19: a cross-sectional study in Japan

**DOI:** 10.1186/s12889-023-16159-9

**Published:** 2023-06-27

**Authors:** Kana Fujii, Hideki Hashimoto

**Affiliations:** Department of Health and Social Behavior, The University of Tokyo School of Public Health, 7-3-1 Hongo, Bunkyo-ku, Tokyo, 113-0033 Japan

**Keywords:** COVID-19, Anticipated stigma, Perceived risk, Normative beliefs, Japan

## Abstract

**Background:**

The stigmatization against COVID-19 has become a public issue. However, it remains unknown which individual factor contributes to anticipated stigma formation. This study explored socio-psychological factors associated with anticipated stigma toward coronavirus disease 2019 (COVID-19).

**Methods:**

We obtained cross-sectional data regarding 1,638 middle-aged community residents (mean age, 48.5 years) from a population-based survey in metropolitan Tokyo, Japan during the third wave of the COVID-19 pandemic, when a regional public health emergency had been declared in December 2020 and January 2021. We hypothesized that perceived risk of infection, normative beliefs about preventive behaviors, and past experiences of stigmatization unrelated to COVID-19 would be associated with anticipated stigma. Modified Poisson regression was conducted to examine associations after adjustments for demographic and socioeconomic statuses.

**Results:**

Higher perceived risk (adjusted prevalence ratio [APR] = 1.17; 95% confidence interval [CI, 1.08–1.27]), past experiences of stigmatization (APR = 1.09; 95% CI [1.00–1.19]), and higher normative beliefs about preventive behaviors (APR = 1.18; 95% CI [1.11–1.26]) were independently associated with anticipated stigma.

**Conclusions:**

These results suggest that intervention messages to increase risk perception and normative beliefs to enhance protective behaviors may have the unintended effect of increasing anticipated stigma in the context of the COVID-19 pandemic.

**Supplementary Information:**

The online version contains supplementary material available at 10.1186/s12889-023-16159-9.

## Background

The coronavirus disease 2019 (COVID-19) pandemic has become a serious social and psychological threat to all people, regardless of their infection status [1, 2]. There have been multiple reports regarding the increasing prevalence of loneliness, anxiety, and psychological stress amidst the pandemic-related uncertainty, lack of information, and restrictions on daily life caused by public health emergency declarations [3, 4]. Some of these reports have addressed the disruption of social relationships because of stigma toward individuals with COVID-19 and those close to them [5–10].

Stigma refers to an attribution of devalued social identity to a person in a particular context, which leads to discrimination against him/her [11, 12]. According to the attribution theory framework, health consequences that are considered preventable/manageable, such as obesity and lifestyle-related diseases, can be a source of stigmatization [13]. Most recently, individuals currently or previously infected with COVID-19 have been targets of social stigmatization and subsequent discrimination [5–10, 14]. The impact of COVID-19-related stigma has also been extended to individuals without the disease [14]. Anticipated stigma is the expectation and fear of stigmatization [15, 16]. It has been argued that people may fear being stigmatized and subjected to discrimination if they become infected with COVID-19 because of prevalent stereotypes that viral contagion results from the “immoral” and/or “irresponsible” personality traits of infected individuals [14]. A recent survey indicated that anticipated stigma related to COVID-19 is prevalent in the general population [14].

Previous studies of anticipated stigma related to psychiatric and other stigmatized conditions have revealed its negative effects on psychological distress, [17, 18] as well as inappropriate behavioral reactions (e.g., fewer interactions with others) [19–21] and the refusal of diagnosis in an effort to avoid the devalued label [14, 22, 23]. These findings strongly suggest that anticipated stigma toward COVID-19 can be a threat to effective case investigation and infection control. Specifically, in addition to the impact of anticipated stigma on psychological well-being, individuals who anticipate stigma tend to conceal their infection status to avoid stigmatization and subsequent discrimination.

Other previous studies have demonstrated that anticipated stigma is associated with the perceived risk of being stigmatized [24] and with past experiences of stigmatization [25]. Additionally, because individuals must manage their own presence based on how they expect others to recognize their own status, [11] anticipated stigma is presumed to result from a normative attitude toward stigmatized status [24]. Thus, an individual’s beliefs about surrounding others’ normative attitude towards compliance with infection control behaviors may influence their expectations of stigmatization [24]. Accordingly, individuals who believe that others around them have strict norms concerning preventive behaviors would be likely to anticipate stigmatization if they get infected because of failures to properly adopt the norm. However, to our knowledge, there is minimal information concerning the factors that shape anticipated stigma related to COVID-19.

In the present study, we hypothesized that factors associated with anticipated stigma include an individual’s perception of infection risk, the past experiences of stigmatization, and perception of normative beliefs related to COVID-19. We believe that the identification of factors associated with anticipated stigma will provide important implications for designing public health messages to promote behavioral responses that facilitate effective infection control, while avoiding social stigma.

## Methods

### Data source

We conducted a secondary data analysis using data derived from the Japanese Stratification, Health, Income, and Neighborhood (J-SHINE) study, a population-based survey, which has been described in detail elsewhere [26]. Briefly, the baseline survey in 2010 involved a probabilistic sampling of adults aged 25–50 years in four metropolitan cities in Japan; respondents were followed up in 2012 and 2017. Data used in the present study were collected in a supplemental survey to assess the health and socioeconomic impacts of COVID-19, which was conducted between December 2020 and January 2021 when the third wave of the pandemic was occurring in the greater Tokyo metropolitan area; at that time, a regional public health emergency had been declared [27]. Survey invitations were sent to respondents whose contact information had been obtained from the most recent 2017 follow-up (*N* = 3,062); 1,638 individuals (53.5%) responded to the online or paper-based questionnaire. There were no significant differences between respondents and non-respondents regarding age, work status, and cohabitant status. The proportion of female respondents was higher than that of female non-respondents in this study (56.0% vs. 52.5%, *p* = 0.05). Respondents were also more likely to have reached an education level higher than university degree compared with non-respondents (46.2% vs. 39.2%, *p* < 0.001). Compared with the first survey in 2010 (*N* = 4,386), the overall response rate was 37.2%. Supplementary Tables 1, Additional File 1 shows the distribution of participants’ age, sex, and educational attainment across each wave to demonstrate the attrition patterns.

### Measures

#### Outcome: anticipated stigma

Because a standardized measurement was unavailable during the survey, anticipated stigma specific to COVID-19 was measured using the following single-item question: “How much do you worry that you and your family will be socially excluded and/or discriminated against if you develop COVID-19?” Responses were measured using a 5-point Likert scale, as follows: “not at all,” “very little,” “neutral,” “some,” and “very much.” In this study, we created a binary variable where “some” and “very much” were regarded as “1”; all other responses were regarded as “0.”

#### Perceived risk of being infected

Perceived risk of COVID-19 was measured with reference to an index used in a previous study to inquire about perceived risk of severe acute respiratory syndrome [28]. The question in the present study was: “How likely do you think you are to develop COVID-19?” and “How likely do you think it is that you will develop COVID-19, compared to other people?” Responses were measured using a 5-point Likert scale, as follows: “not at all,” “very little,” “neutral,” “some,” and “very much.” The mean score for the items exhibited a Cronbach’s alpha of 0.82 in our study sample [29]. The combined score was dichotomized using the median value of 3.5 as the cutoff point; responses below this cutoff were regarded as “0” and considered indicative of low perceived risk, while responses above this cutoff were regarded as “1” and considered indicative of high perceived risk.

#### Comorbidity status susceptible to stigmatization

Because the survey did not directly assess each respondent’s past experiences of stigmatization, we used specific comorbidity statuses as proxy measures for the likelihood of past experiences of stigmatization, based on the findings in previous studies. We presumed that individuals with chronic diseases (e.g., diabetes mellitus, [30–32] psychiatric disorders, [33, 34] arthritis/rheumatoid arthritis, [35, 36] and chronic respiratory diseases [37]) had a high likelihood of previous stigmatization.

#### Normative beliefs concerning protective behavior

Normative beliefs concerning protective behavior were measured by using a three-item 5-point Likert scale questionnaire described in previous studies [38, 39]. The first two items asked if the respondents believed that persons important to them (e.g., family, close friends) would expect them to take appropriate protective action against COVID-19 infection, and the third item asked about respondents’ motivation to comply with the expectations of their important persons. In accordance with the original recommendations for the treatment of a normative belief scale in the theory of planned behavior [40], we scored the scale by taking the average score of the first two items (related to subjective normative beliefs) multiplied by the score of the third item (related to compliance to the surrounding norms). Additionally, we scaled scores by taking the average score on the three items (for which Cronbach’s alpha was 0.87). This rescaling did not substantially affect the results. A higher score was considered indicative of stronger normative beliefs concerning behavioral compliance.

#### Covariates

In addition to age, sex, and level of education, respondents’ work status and cohabitation status were included as covariates in the analysis model because people who have jobs or who live with others in the same household were presumed to perceive a greater likelihood of developing COVID-19 or transmitting the infection to others in their workplaces and/or households.

### Statistical analysis and ethical considerations

After summarizing the data using descriptive statistics, we conducted modified Poisson regression analysis [41] with anticipated stigma as a dependent variable; perceived risk, normative belief, and comorbidity status likely to experience stigma were regarded as major independent variables, with adjustments for age, sex, education, job status, and cohabitant status.

We conducted hierarchical modeling where Model 1 was adjusted for the covariates of age, sex, education, work status, and cohabitation status. In Model 2, in addition to the Model 1 covariates, we conducted separate analyses including, respectively, perceived risk, comorbidity status susceptible to stigmatization, or normative beliefs independently. Finally, Model 3 included all above variables. All *p*-values were two-sided. We used Stata/SE 16.1 (StataCorp, College Station, TX, USA) for all statistical analyses.

The study participants expressed their consent to participate in the study through online or written consent forms, and the survey was approved by the internal review board in the authors’ affiliated institution (IRB number 2020231NI).

## Results

### Participant characteristics

Table 1 shows the descriptive statistics of the participants. High anticipated stigma was present in 1,084 participants (66.3%). Compared with the low anticipated stigma group, participants in the high anticipated stigma group were more likely to be female, younger, have a lower level of education, live with others, have a higher perceived risk of being infected, and have a comorbidity status susceptible to stigmatization. Additionally, this group had a higher mean score of normative beliefs concerning protective behaviors. Work status was not associated with anticipated stigma. Two (0.12%) of the participants reported previous diagnoses of COVID-19 (data not shown in the table). At the time the survey was conducted, the cumulative number of COVID-19 cases in Japan was approximately 200,000 (0.16% of the total population).

**Table 1:** Characteristics of the study participants

	Anticipated stigma		
	High	Low	p^c^
	*n*=1,084	*n*=545	
	N (%) or Mean (± SD)	N (%) or Mean (± SD)	
Sex			
Male	420 (38.7)	289 (53.0)	<0.001
Female	664 (61.3)	256 (47.0)	
Age, years	48.1 (± 7.0)	49.3 (± 7.2)	0.001
Education			
Lower than university degree	610 (56.6)	262 (48.2)	0.001
University degree or higher	468 (43.4)	282 (51.8)	
Work status			
Employed	952 (87.9)	482 (89.0)	0.545
Unemployed	131 (12.1)	60 (11.1)	
Cohabitation status			
Living with others	919 (91.7)	450 (88.8)	0.061
Living alone	83 (8.3)	57 (11.2)	
Perceived risk^a^			
High	676 (62.5)	270 (49.6)	<0.001
Low	405 (37.5)	274 (50.4)	
Comorbidity status susceptible to stigmatization^a^			
Yes	194 (18.0)	75 (13.8)	0.034
No	890 (82.1)	470 (86.2)	
Diabetes mellitus			
Yes	43 (4.0)	13 (2.4)	0.098
No	1,041 (96.0)	532 (97.6)	
Psychiatric disorder			
Yes	66 (6.1)	32 (5.9)	0.862
No	1,018 (93.9)	513 (94.1)	
Arthritis/rheumatoid arthritis			
Yes	42 (3.9)	17 (3.1)	0.441
No	1,042 (96.1)	528 (96.9)	
Chronic respiratory disease			
Yes	63 (5.8)	25 (4.6)	0.302
No	1,021 (94.2)	520 (95.4)	
Strong normative beliefs^b^	18.3 (± 4.7)	16.9 (± 4.9)	<0.001

*SD* Standard deviation

^a^Cronbach’s alpha: perceived risk (0.82), norm (0.87)

^b^Possible score ranging from 1 to 5

^c^Student’s t-test for continuous variables and Pearson’s chi-squared test for categorical variables.

### Associations of individual factors with anticipated stigma

Table 2 presents the results of the modified Poisson regression analysis. Model 1 showed that women, younger individuals, and less educated individuals had higher perception of anticipated stigma. Work status and cohabitation status were not associated with the prevalence of high anticipated stigma. Model 2 revealed that high perceived risk of disease, comorbidity status susceptible to stigmatization, and strong normative beliefs concerning preventive behaviors were all associated with high anticipated stigma, independent of the baseline variables in Model 1. The addition of these variables did not substantially affect the coefficients of baseline variables in Model 1. The inclusion of all three variables (Model 3) showed that perceived risk (adjusted prevalence ratio (APR) = 1.17, 95% confidence interval (CI) [1.08–1.27]), comorbidity status susceptible to stigmatization (APR = 1.09, 95% CI [1.00–1.19]), and strong normative beliefs (APR = 1.02, 95% CI [1. 01–1.03]) remained positively associated with anticipated stigma. When we included each of the comorbidity conditions separately in Model 3, the association between diabetes and anticipated stigma remained (APR = 1.22, 95% CI [1.04–1.43]), but there was no association between psychiatric disease and anticipated stigma (APR = 0.97, 95% CI [0.83–1.14]). The coefficients of these variables were only slightly attenuated, suggesting that they were independently associated with higher perception of anticipated stigma after adjustments for each other.

**Table 2:** Results of hierarchical Poisson regression to predict anticipated stigma

	Model 1		Model 2 Perceived risk		Model 2 Comorbidity status likely to experience stigma		Model 2 Norm		Model 3	
	PR	95% CI	PR	95% CI	PR	95% CI	PR	95% CI	PR	95% CI
Sex										
Female	1.17	(1.08–1.27)	1.17	(1.08–1.26)	1.17	(1.08–1.27)	1.16	(1.07–1.26)	1.17	(1.08–1.26)
Male	ref		ref		ref		ref		ref	
Age, years	0.99	(0.99–1.00)	0.99	(0.99–1.00)	0.99	(0.99–1.00)	0.99	(0.99–1.00)	0.99	(0.99–1.00)
Education										
Lower than university degree	1.09	(1.01–1.18)	1.10	(1.02–1.18)	1.09	(1.01–1.18)	1.10	(1.02–1.19)	1.10	(1.02–1.19)
University degree or higher	ref		ref		ref		ref		ref	
Work status										
Employed	1.05	(0.94–1.17)	1.00	(0.89–1.13)	1.05	(0.94–1.18)	1.02	(0.91–1.14)	0.98	(0.87–1.10)
Unemployed	ref		ref		ref		ref		ref	
Cohabitation status										
Living with others	1.09	(0.95–1.25)	1.08	(0.94–1.24)	1.09	(0.95–1.26)	1.07	(0.93–1.23)	1.07	(0.93–1.23)
Living alone	ref		ref		ref		ref		ref	
Perceived risk of being infected										
High			1.19	(1.10–1.29)					1.17	(1.09–1.27)
Low			ref						ref	
Comorbidity status susceptible to stigmatization										
Yes					1.11	(1.01–1.21)			1.09	(1.00–1.19)
No					ref				ref	
Normative beliefs concerning protective behavior							1.02	(1.01–1.03)	1.02	(1.01–1.03)

*PR* Prevalence ratio, *CI* Confidence interval

## Discussion

To our knowledge, this is the first study to empirically investigate the socio-psychological factors associated with anticipated stigma related to COVID-19. We found that more than half of the respondents (66%) had a high level of anticipated stigma during the pandemic wave when a public health emergency was declared in metropolitan Tokyo. We also found that risk perception, comorbidity status susceptible to stigmatization, and strong normative beliefs were all positively associated with high anticipated stigma; each explanatory variable was independently associated with anticipated stigma.

A previous study revealed associations between perceived risk and anticipated stigma among individuals with a high risk of human immunodeficiency virus (HIV) infection [24]. The present study demonstrated a similar association with respect to COVID-19, but careful comparisons are needed. Since the likelihood of HIV infection was known to relate to the degree of risky sexual behavior, the social stigma toward individuals with HIV infection was regarded as the mechanism that linked one’s risk perception and anticipated stigma [24]. In contrast, perceived risk concerning COVID-19 was positively associated with the likelihood of engaging in prevention behaviors such as social distancing and mask wearing, while it was also associated with older age and comorbidities that increased the risk of severe pneumonia [42]. Thus, despite differences in the composition of risk perceptions and related behaviors, perceived risk demonstrated a similar association with anticipated stigma, presumably because of the underlying social stigma toward individuals with COVID-19.

The present results are not inconsistent with the findings in previous studies, which showed that individuals who had experienced stigma were more likely to have anticipated stigma [25]. Because stigma causes social and psychological distress [43, 44] to individuals who experience it, those individuals may be more sensitive to encountering similar experiences in the future, even with different diseases. In particular, diabetes is widely acknowledged to be a strong risk factor for severe COVID-19-related complications [45], which may explain why, of the comorbidities, diabetes showed the strongest relationship with anticipated stigma.

Finally, the present findings support the idea that normative beliefs concerning preventive behaviors lead to greater anticipated stigma; this association was independent of perceived risk and past experiences of stigmatization. Previous studies showed that the perception of stigma varies according to the perception of the surrounding social environment; individual’s expectations about how others are likely to perceive that individual can also contribute to the stigmatized status [46, 47]. With respect to the COVID-19 pandemic, the norm for the strict compliance with protective behaviors may lead to disease-related stigmatization because the disease development is likely taken as a consequence of non-compliance with the norm. Under these circumstances, perceived risk may encourage engagement in preventive behaviors while increasing the sense of anticipated stigma.

### Implications

The results of this study have important implications for public health. First, the finding that 66% of community survey respondents had a high level of anticipated stigma suggests that psychological problems related to anticipated stigma [17, 18] and potential test refusal [14] may be issues that cannot be overlooked in efforts to achieve successful infection control. These results strongly suggest a need for careful design of public health messages to protect people from anticipated stigma.

The finding that increasing risk perception and normative beliefs may lead to greater anticipated stigma is a major challenge and dilemma for public health intervention; perceived risk and strong normative beliefs are known to enhance behavioral intentions for COVID-19 prevention measures [48–52]. A previous study also claims that educating about the correct knowledge about COVID-19 increases anxiety, but this has the benefit of promoting preventive behavior against infection [53]. However, our results suggest that public health messages designed to increase normative beliefs may have the unintended effect of increasing anticipated stigma. The establishment of balance between the positive and negative effects of normative belief formation on infection control is beyond the scope of the present study; further theoretical and empirical research is warranted.

### Limitations

There were several limitations in this study. First, this study used cross-sectional data, and causality cannot be inferred from the results. In this survey, the regression results indicated that women were more vulnerable to anticipated stigma than men. However, because women were overrepresented in our sample owing to selective attrition of male respondents, we may have overestimated the sex differences. Another problem was that the survey non-respondents were more likely to have education lower than university degree, which may have led to underestimation of the effect of low educational attainment on anticipated stigma. In addition, considering the age structure of the participants in 2010, it is possible that the effect of age on anticipated stigma was underestimated, as there would have been fewer young people in 2020. Second, past experiences of stigmatization were not directly examined; they were assumed based on a proxy measure of diseases with high likelihood of experiencing stigma. Therefore, some people who did not experience stigma might have been incorrectly included in the group of participants who experienced stigma. Such misclassification would weaken the observed association between experiences of stigmatization and the perception of anticipated stigma. Additionally, previous studies have shown that individuals with ulcerative colitis [54, 55] and acquired immunodeficiency syndrome [56, 57] are more likely to experience stigma; however, we did not consider these diseases in our analysis because the necessary information was not collected in the survey. Third, those who are indifferent to COVID-19 may have been reluctant to complete this survey, because it is designed as a survey measuring the impact of COVID-19. Finally, the survey was conducted in the middle of the third wave of COVID-19 in Japan and there was general compliance with prevention guidance. Further research is needed to determine whether the widespread occurrence of anticipated stigma was an effect of this specific stage of the pandemic.

## Conclusions

In this study, we examined individual factors associated with anticipated stigma toward COVID-19. We found that risk perception, past experiences of stigmatization, and strong normative beliefs were independently associated with anticipated stigma. These results suggest that interventions to increase risk perception and normative beliefs may have the unintended effect of increasing anticipated stigma. In the context of the COVID-19 pandemic, there is a need to identify effective design of infection control messages that avoid anticipated stigma.

## Supplementary Information

**Additional file 1: Supplementary Table 1.** Age, sex, and educational attainment in J-SHINE waves of 2010, 2017, and 2020.

## Data Availability

J-SHINE data will be made available after approval for data use by the J-SHINE data management committee.

## Abbreviations

COVID-19: Coronavirus disease
WHO: World Health Organization
