# Supplementary material for: Socio-psychological factors associated with anticipated stigma toward COVID-19: a cross-sectional study in Japan
Source: BMC Public Health. 2023 Jun 27;23:1245. doi: 10.1186/s12889-023-16159-9 (PMC10294524; doi:10.1186/s12889-023-16159-9)
Supplement: Supplementary file 1 — Additional file 1: Supplementary Table 1. Age, sex, and educational attainment in J-SHINE waves of 2010, 2017, and 2020. [file 12889_2023_16159_MOESM1_ESM.docx]

Supplementary Table 1. Age, sex, and educational attainment in J-SHINE waves of 2010, 2017, and 2020

|  |  | 2010 (n=4,385)  N (%) | 2017 (n=2,787)  N (%) | 2020 (n=1,638)  N (%) |
| --- | --- | --- | --- | --- |
| Sex | |  |  |  |
|  | Male | 2,043 (46.6) | 1,233 (44.2) | 721 (44.0) |
|  | Female | 2,342 (53.4) | 1,554 (55.8) | 917 (56.0) |
| Age, years (as of 2010) | |  |  |  |
|  | 25–30 | 980 (22.4) | 512 (18.4) | 292 (17.8) |
|  | 31–40 | 1,781 (40.6) | 1,133 (40.6) | 679 (41.5) |
|  | 4150 | 1,624 (37.0) | 1,142 (41.0) | 667 (40.7) |
| Education | |  |  |  |
|  | Lower than university degree | 2,441 (56.2) | 1,588 (57.3) | 878 (53.8) |
|  | University degree or higher | 1,905 (43.8) | 1,184 (42.7) | 753 (46.2) |
